# Culturally and linguistically appropriate vaccine education (CLAVE) for Indigenous communities: design and implementation study of a community-based approach to digital vaccine education in the Central Highlands of Guatemala

**DOI:** 10.1186/s12939-026-02836-9

**Published:** 2026-04-14

**Authors:** Jamie Sewan Johnston, Lucia Abascal Miguel, Rubi Gaitán Barrillas, Emily Lopez, Julia Coxaj, Magda Silvia Sotz Mux, Parminder S. Suchdev, Jose Carlos Monzon Fuentes, Esbeydy Pardo, Semay Johnston, Nadine Ann Skinner, Anne Kraemer Diaz, Nadia Diamond-Smith, Victoria C. Ward

**Affiliations:** 1 Stanford Center for Health Education, Stanford University School of Medicine, Stanford, CA USA; 2 Institute for Global Health Sciences, University of California, San Francisco, San Francisco, CA USA; 3 Wuqu´ Kawoq, Maya Health Alliance, Tecpán, Guatemala; 4 Centers for Disease Control and Prevention, Central America, Guatemala City, Guatemala; 5 Department of Epidemiology and Biostatistics, University of California, San Francisco, San Francisco, CA USA; 6 Department of Pediatrics, Stanford University School of Medicine, Stanford, CA USA

**Keywords:** Community health workers, Indigenous health, Vaccine education, Routine immunization, Human papillomavirus (HPV), Social media, Digital health education, Human-centered design

## Abstract

**Background:**

In Guatemala, childhood vaccination rates, particularly in Indigenous communities, are low due to inaccessible information and mistrust. Using a human-centered design (HCD) process with community partners, we developed CLAVE (Culturally and Linguistically Appropriate Vaccine Education), a video series on routine and human papillomavirus (HPV) immunization in Kaqchikel, K’iche’, and Spanish. Content was distributed via social media and through community health workers (CHWs). This study describes the instructional design process and assesses implementation feasibility through community-level measures of reach and engagement and acceptability among CHWs across 12 communities in the Central Highlands of Guatemala.

**Method:**

To develop content, we applied an HCD framework and collected iterative feedback from communities through 16 CHW interviews and nine focus group discussions with 42 community members. We implemented CLAVE in 12 communities, with four communities randomly assigned in parallel to each arm: (1) a six-week, geographically targeted social media campaign promoting CLAVE videos; (2) the same social media campaign plus CLAVE videos and complementary digital infographic job aids provided to CHWs; and (3) a control arm with no exposure to CLAVE videos or the CHW intervention. Platform analytics were used to assess the reach and engagement potential on social media, and CHW survey data were used to assess acceptability of content among CHWs.

**Results:**

The CLAVE campaign reached 486,594 unique social media platform users, with over 4.1 million impressions and a high overall engagement rate reflecting multiple user interactions with content. Spanish and Kaqchikel versions reached the largest audiences, while K’iche’ content generated proportionally higher engagement and viewing rates, suggesting stronger interaction among smaller language audiences. Vaccine acceptance was high across all CHWs. Amid a high baseline, the intervention was not associated with significant differences in CHWs’ perceptions of the efficacy or importance of routine childhood vaccination or HPV vaccination, nor with knowledge measures. However, CHWs receiving CLAVE content directly were more likely to strongly agree that the HPV vaccine is safe (*p* < 0.05). CHWs reported high perceived usefulness and frequent utilization of CLAVE content.

**Conclusions:**

This pilot suggests that geographically targeted paid social media distribution of community-engaged, multilingual vaccine education content can achieve broad exposure, and that pairing videos with job aids can support CHW vaccine education efforts.

**Registry:**

ClinicalTrials.gov, TRN: NCT06186206, Registration date: 05 January 2024.

**Supplementary information:**

The online version contains supplementary material available at 10.1186/s12939-026-02836-9.

## Background

Historically, Guatemala has experienced the lowest routine vaccination coverage in Latin America, and coverage further declined due to recent setbacks driven by the COVID-19 pandemic [1–4]. Routine immunization coverage for children ages 12 months and older remains notably deficient, as families often fail to complete the full vaccination schedule [1]. Between 2019 and 2022, the percentage of children who received three doses of the vaccine against diphtheria, tetanus, and pertussis (DTP3) in order to achieve full immunity fell six percentage points to 79% nationally, while the percentage of children receiving their second dose against Measles, Mumps, and Rubella (MMR2) fell nine percentage points to 69%, both rates well below the 95% regional target set by the Pan American Health Organization [2, 5]. Likewise, vaccine coverage of the human papillomavirus vaccine (HPV) is alarmingly low, at 18% in 2022, falling from 32% in 2018, driving high rates of cervical cancer, the second most common cancer among women aged 15 to 44 in Guatemala [2, 6–9].

While vaccine coverage is low nationwide, uptake is especially low in rural and mainly Indigenous communities, where vaccine hesitancy is fueled by a lack of accessible health information in Mayan languages and mistrust of the health system [10, 11]. Over 40% of Guatemala’s population identifies as Indigenous and collectively speaks over 20 non-Spanish languages [12]. Outside of Guatemala City, the departments (Guatemalan administrative divisions equivalent to states or provinces) with the highest percentage of Indigenous residents also tend to have the lowest estimates of vaccine coverage [13–15]. During the COVID-19 pandemic, Indigenous communities in Guatemala were disproportionately burdened [16], with evidence pointing to a significant gap in culturally and linguistically appropriate health education to respond to their needs [10, 17].

The situation for Indigenous communities in Guatemala is not unique. Worldwide, access to basic healthcare provision is often challenging for those who speak Indigenous languages, and Indigenous language-speaking communities are often not prioritized in the development of health interventions [18]. Global research has shown that Indigenous communities, as well as other populations living in areas where they do not speak the dominant language, experience difficulty navigating healthcare facilities [11, 19, 20], are more frequently misdiagnosed [20–22], and suffer from adverse health impacts [23–25] including higher rates of disease and difficulty managing treatment recommendations [19, 20, 26–30]. Among these populations, fear and distrust of health systems and healthcare facilities are high due to sociocultural and linguistic barriers [22, 31–34].

Beyond barriers associated with language, Indigenous communities face a legacy of historical traumas, including exploitation in medical settings that compounds the challenges for the promotion of vaccine acceptance [35–37]. In Guatemala, the enduring effects of colonialism, state-sponsored violence, and marginalization have set the stage for the systematic exclusion of Indigenous populations from the healthcare system, which is reflected in the inequitable health infrastructure and education available to rural Indigenous communities, as well as their overwhelming mistrust in health information provided by the government and health facilities [11, 38–41].

Across health systems in low- and middle-income countries (LMICs), community health workers (CHWs) often serve as the first point of care and information delivery to communities with limited access to basic health information, including guidance on routine childhood immunization [42, 43]. When adequately trained, equipped, and supported, CHWs play a critical role in improving maternal and child health and reducing infant and under-five mortality and morbidity [44–47]. In Guatemala, findings suggest that one of the most effective strategies to promote vaccination and combat local mis- and disinformation is to equip CHWs, who are widely regarded as trusted sources of information in their communities, with training and resources to deliver accurate information and dispel prevalent myths [10].

As the COVID-19 pandemic amplified public mistrust, myths, and misinformation about vaccination, undermining confidence in immunization efforts [48], social media platforms have served as conduits for the rapid and widespread dissemination of false and misleading information about vaccinations [49–51]. Nevertheless, social media can be leveraged as part of the solution, with some success, albeit limited, demonstrated by health education campaigns to influence perceptions around vaccination [51]. Evidence suggests that campaigns are more likely to achieve success when they deliver tailored, culturally relevant messages through trusted messengers, including in Guatemala [52], but more research is needed to determine effective strategies for scale [51].

To address these gaps [53], the Stanford Center for Health Education’s Digital Medic initiative, the Institute for Global Health Sciences at the University of California, San Francisco, and the Guatemalan health non-governmental organization (NGO), Wuqu’ Kawoq | Maya Health Alliance with support from the Guatemalan Ministry of Health collaborated to develop and pilot a vaccine education series that prioritizes the experiences and needs of Indigenous Maya communities, entitled CLAVE (Culturally and Linguistically Appropriate Vaccine Education). CLAVE is a vaccine education video series, with complementary CHW job aids designed for communication with and distribution amongst mainly Indigenous rural communities.

The intervention was initially conceptualized as a geographically targeted social media campaign to disseminate culturally and linguistically tailored vaccine education content. During the human-centered design process in which we engaged to develop content, community stakeholders highlighting the importance of supporting CHWs with materials to support in-person education and discussions with families. Based on this feedback, the intervention evolved to include a complementary CHW delivery pathway, including CLAVE videos and infographic job aids designed for use during community interactions.

This paper describes the human-centered instructional design process used to develop the CLAVE intervention and reports findings from a pilot implementation. Specifically, we aimed to (1) document the participatory design process used to develop culturally and linguistically appropriate vaccine education content, (2) examine implementation feasibility through measures of social media reach and engagement, and (3) assess CHW acceptability and perceived usefulness of the CLAVE materials. We also explored changes in CHW knowledge and perceptions related to vaccination.

## Methods

### Human-centered design

To develop CLAVE, we utilized a community-engaged education design process grounded in human-centered design (HCD) principles [54, 55]. We turned to HCD principles to respond to the multifaceted and nuanced nature of vaccine hesitancy in Indigenous communities [56–58]. As defined by the International Organization for Standardization, HCD is an approach to design that prioritizes user empathy and user involvement with ideation, prototyping, and iteration to ensure the alignment of the final product with user needs (ISO 9241–210). While HCD originated in computer science and engineering product design, it has been increasingly applied to global health initiatives [55], with demonstrated success in improving maternal child health practices [59–64]. HCD approaches have also been used in a range of interventions aimed at promoting vaccine acceptance [63, 65–68].

**Fig. 1:**
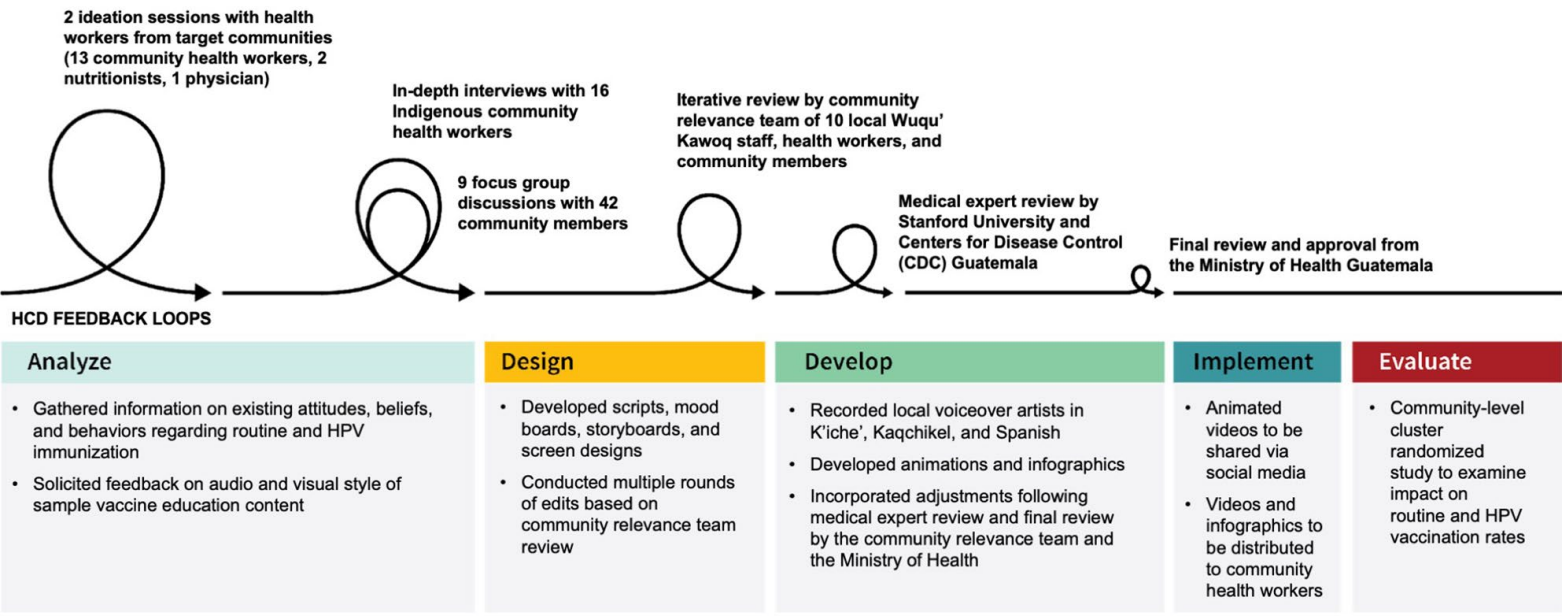
CLAVE human-centered (HCD) instructional model following ADDIE framework

Specifically, we followed the ADDIE (Analyze, Design, Develop, Implement, and Evaluate) instructional design framework [69, 70], involving a series of target-user feedback loops with CHWs and the members of the communities they serve in the Central Highlands of Guatemala. From the initiation of the project, we involved the leadership, participation, and iterative feedback of CHWs and community members to provide key vaccine information to speakers of the two most widely spoken Mayan languages in Guatemala, K’iche’ and Kaqchikel [14]. Prototype materials were repeatedly reviewed and revised based on feedback, allowing community perspectives to shape key decisions related to messaging, language, and visual design throughout the development process. Fig. 1 illustrates our design process with feedback loops starting with higher-level ideation and narrowing as design decisions were made with the iterative feedback provided by key target audience members and other relevant stakeholders, including representatives from the Centers for Disease Control & Prevention (CDC) in Guatemala and the Guatemalan Ministry of Health.

Our first design phase, “Analyze,” began with higher-level ideation through two hour-long content design sessions with Indigenous health workers from the target communities, including 13 CHWs, two nutritionists, and one physician. In the ideation sessions, participants were asked to provide information on current vaccine campaigns and identify key messages and important information for the design of content for Indigenous Maya communities. Participants were also asked to provide feedback on the visual and audio styles for content design. Following feedback from the ideation sessions, the Stanford medical expert and instructional design team developed design documents in English and Spanish, identifying key learning objectives, sample content based on these ideation sessions, and qualitative data collection tools for further community input.

We then conducted in-depth interviews (IDIs) with 16 CHWs and 9 focus group discussions (FGDs) involving 42 community members. FGDs were conducted in gender-segregated groups to promote participant comfort and open discussion. The IDIs inquired about CHW perspectives on sources of vaccine hesitancy, trustworthy sources of information sharing, and reactions to educational messaging in the communities they serve. The FGDs delved into beliefs, attitudes, and behaviors regarding immunizations and their trusted sources of health information. IDIs and FGDs also solicited feedback on prototype content developed from ideation sessions.

The Wuqu’ Kawoq team recruited all participants through their community networks. To engage a broader set of participants, the sample was expanded through a snowball technique. Local Guatemalan team members conducted interviews and focus group discussions in Spanish, K'iche', or Kaqchikel, recorded audio, and transcribed the audio recordings. Transcripts were checked for accuracy and translated into Spanish (when applicable) and English for analysis.

Using inductive and deductive methods, the research team conducted a rapid analysis of transcripts to inform and expedite the development of content. Two reviewers coded the transcripts independently. Employing constant comparative methods, we ensured the validity of the code application, performed reliability checks, and identified primary themes and key messages. The larger research team convened to reach a consensus on the themes and messages, which were then presented to local interviewers for additional validation.

**Fig. 2:**
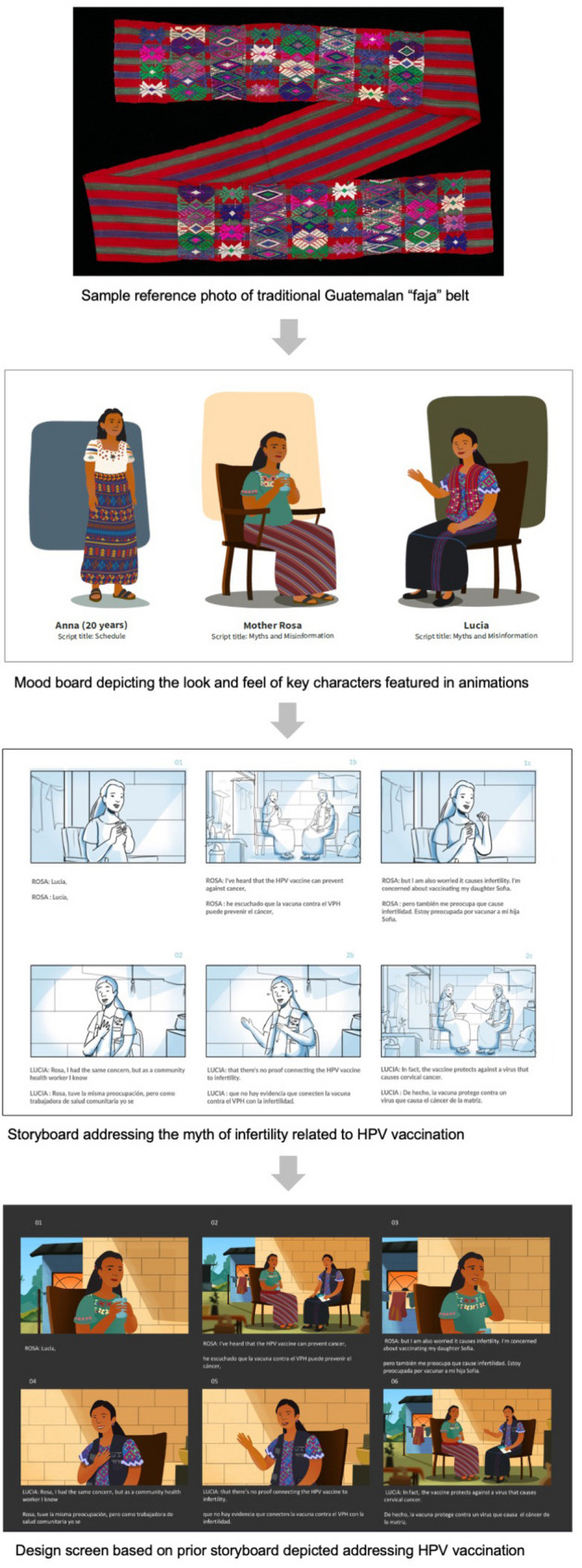
Sample CLAVE mood board, storyboard, and design screen

The primary themes and key messages were relayed to the instructional design team, commencing the second “Design” phase in which the design team engaged in the development of scripts following feedback from communities and established best practices in line with guidelines from the Guatemalan Ministry of Health, the Centers for Disease Control and Prevention (CDC), and the World Health Organization. As depicted in Fig. 2, the instructional design team developed mood boards, drawing inspiration from real-life images. A team of 10 local Wuqu’ staff members, Indigenous health workers, and community members was assembled to provide iterative review through the “Design” and “Develop” phases. Participants were identified through Wuqu’ Kawoq’s community networks based on their experience working with communities in the Central Highlands and their familiarity with local health communication practices. This group reviewed scripts, storyboards, and design screens (as depicted in Fig. 2) and provided iterative feedback on cultural relevance, language, visual style, and messaging through shared documents, email, and regular meetings with the instructional design team.

In the “Develop” phase of content development, the instructional design team developed the full animated video content and infographics. The local team also led the translation and refining of scripts and created voiceover recordings in K'iche', Kaqchikel, and Spanish. The Stanford University team provided medical expert review throughout content development, including original scripting, incorporation of iterative feedback, and image production, and conducted a final review when development was finalized. The CDC in Guatemala also conducted an expert medical review following completion. The community relevance team provided feedback on the final version of CLAVE content, which was then submitted to the Guatemalan Ministry of Health for final review and approval.

While the findings of our full qualitative thematic analysis are described fully in a separate manuscript [71], we report here on three main findings from our HCD process that drove key changes in the development of the CLAVE intervention: (1) Resonance with the audio and visual style of content matters. (2) HPV messaging should be distinct from messaging around other routine childhood immunizations. (3) CHWs are trusted in communities as effective deliverers of health education.

**Fig. 3:**
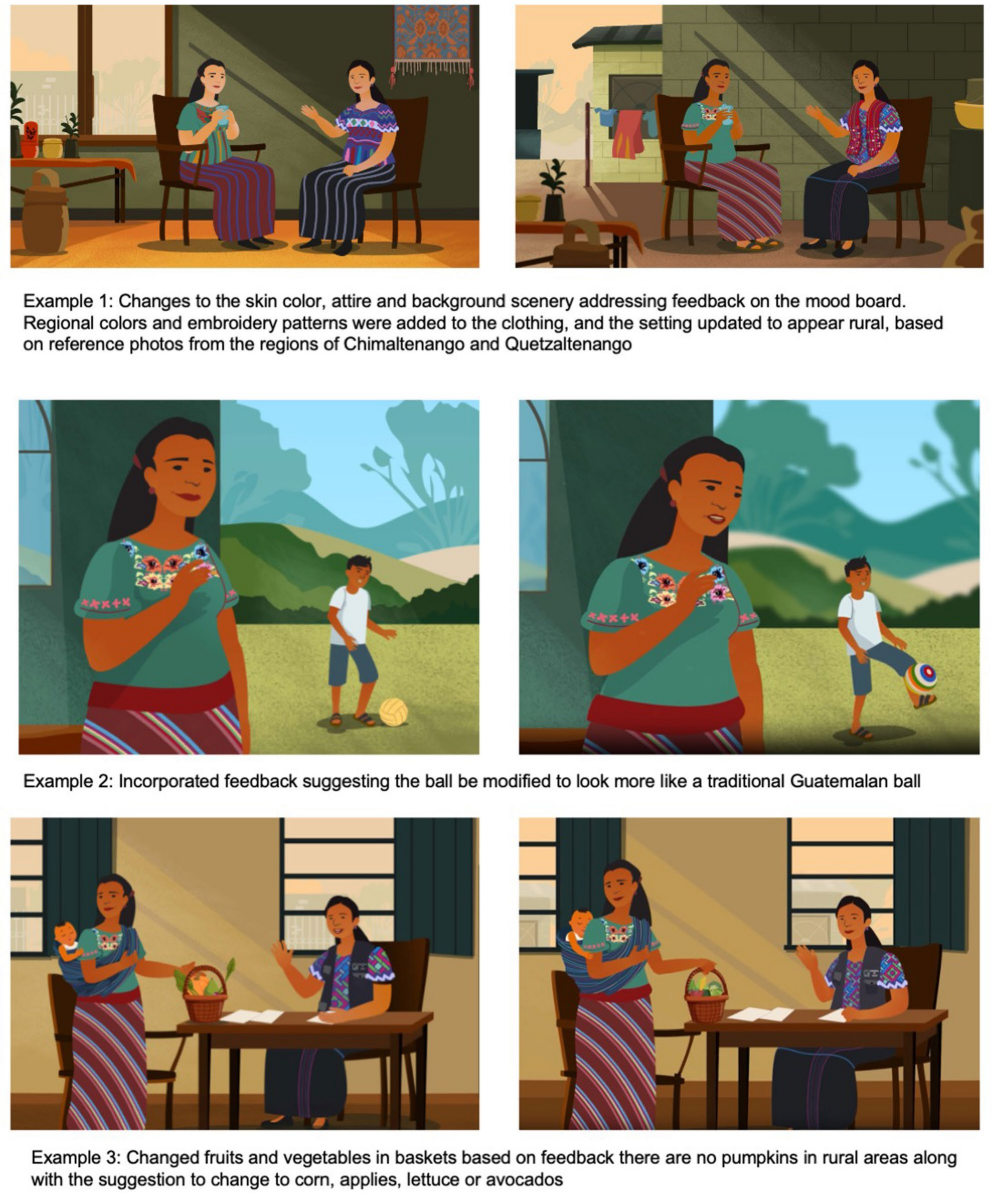
Design screen iteration based on human-centered design (HCD) feedback

First, throughout the HCD process, Indigenous community members overwhelmingly reported the perception that existing vaccine education is not made for them, both regarding availability in preferred languages and cultural and contextual resonance. The lack of resonant information was linked to mistrust of vaccine information. Iteration based on the ADDIE “Design” phase focused on ensuring the look and feel of the animations were appropriate and acceptable for the target communities. Figure 3 displays three examples of modifications made to design screens based on the community review process.

Second, interview and focus group data revealed that CHWs and community members perceive HPV vaccination as different from routine childhood immunization. We observed that in the target communities, HPV vaccination is associated with stigma and distinct myths about the implications for fertility that should be addressed differently from routine immunization. Respondents also revealed a lack of awareness about HPV and its link with cervical cancer. The decision was thus made in the “Design” phase to develop two separate video series that address HPV and routine immunization independently.

Third, the process revealed the trust in CHWs, which led us to design a separate intervention arm that delivers CLAVE videos and complementary infographic job aids directly to CHWs. Community members revealed greater trust in vaccine information delivered from known community sources and particularly CHWs. At the same time, the interviews with CHWs illustrated the need for communication tools that can support their efforts to educate community members about vaccination. For each video series, a digital infographic job aid for CHWs was developed, containing key messages related to routine immunization and HPV immunization, respectively. The full set of CLAVE videos is detailed in the Supplement.

### Study population

For the final ADDIE phases of “implement” and “evaluate”, the Wuqu’ Kawoq team selected 12 communities within the Central Highlands of Guatemala for inclusion in the pilot study. The communities are located in departments designated as priority areas by the Ministry of Public Health and Welfare (MSPAS). Six communities are located in the department of Chimaltenango, three in Sololá, two in Sacatepéquez, and one in Quiche. Per the 2018 census, the estimated population of the communities ranges between approximately 14,000 to 145,000. [14] The communities are primarily rural, and the ethnic majority in each is Maya, with a young demographic including a significant proportion of the population under 14 years of age. While poverty levels are somewhat varied, they are generally high, with several communities facing poverty rates greater than 75%. In three communities, the dominant Mayan language is K'iche', and in the remaining 9 communities, Kaqchikel.[14]

While we do not have a precise measure in the focal communities, internet penetration across Guatemala is over 50% [72], with nearly 8 million Facebook users nationwide, roughly 44% of the population. [73] Of the departments where the communities are located, Facebook penetration is highest in Sacatepéquez at 45%, but lower than a third of the population in the remaining departments [74].

### Study design

The pilot study was designed with three study arms to assess the effectiveness of CLAVE, with four communities randomly assigned in a parallel design to each arm: (1) In the first arm, communities were geographically targeted for a social media campaign promoting CLAVE videos. (2) In the second arm, in addition to receiving the social media campaign, CHWs in those communities were recruited to receive CLAVE videos and complementary digital infographic job aids. (3) The third arm was a control that was not exposed to CLAVE videos or the CHW intervention. We stratified randomization, first allocating the three largest communities to separate arms, then randomly assigning the remaining communities to the three arms.

We used the Facebook ads platform to circulate CLAVE videos in the two treatment arms, which delivers promoted content across both Meta products, Facebook and Instagram. The campaign ran in geographically targeted areas with a radius of 5 miles around the selected communities for six weeks from June to August 2024. Videos were promoted via the Wuqu’ Kawoq Facebook page. CLAVE videos appeared on Facebook and Instagram mobile feeds and in linked browser ads approximately 1–2 times per week during the duration of the campaign in the targeted communities. We applied age targeting to promote videos only to users 18 years or older. In the treatment communities, videos were promoted in Spanish and the dominant Mayan language of the community (K'iche' or Kaqchikel). HPV and routine immunization videos were promoted using the same targeting parameters, and rollout strategy, with no differences in dissemination approach by topic. Wuqu’ Kawoq staff monitored activities and comments on the posts.

To recruit CHW participants, we worked with the leadership of entities (government or non-governmental organizations) designated to supervise the work of CHWs. CHWs involved in the design and development phases were not recruited for the implementation phase. Consenting CHWs in all communities participated in data collection, and in the four treatment communities randomly selected for CHW distribution, we delivered CLAVE videos and the accompanying job aids via the platform, WhatsApp, which was selected as the preferred and established digital communication platform during planning discussions with leadership entities. The Wuqu’ Kawoq team managed WhatsApp communications with participants. All messages were sent individually (one-to-one) to CHWs rather than in group chats. WhatsApp messages containing downloadable video files in all three languages, as well as links to YouTube playlists, were sent to CHWs over 15 days. Along with the videos and links, CHWs received messages encouraging CHWs to view the videos and to share videos with households in their care, including during home visits. CHWs received the same guidance and instructions for using HPV and routine immunization videos, with no differences in recommended use by vaccine type.

The third arm, serving as the control group, consisted of four communities receiving the standard of care in this setting. Roughly six months after the completion of the data collection, we distributed the CLAVE videos via social media and directly to CHWs in all communities. This study was registered on clinical trials (NCT06186206) prior to implementation.

### Data collection

This study draws upon two sources of data to examine the reach and engagement with CLAVE content and to investigate impact on CHW knowledge and perceptions.

1. Social media analytics: Social media performance data were obtained from Facebook Ads Manager, which reports metrics for promoted content across Facebook and Instagram. Following standard metrics of social media performance, we extracted the following variables for the campaign period: reach (unique users who viewed the content), impressions (total displays), engagement (including video plays of three seconds or longer, reactions, comments, shares, saves, and other link clicks), and ThruPlay (indicating a user has watched a video to completion or for at least 15 seconds, whichever comes first) [75]. We calculated engagement and ThruPlay rates by dividing total engagements and ThruPlays by reach, aggregating for the campaign period and stratifying by language and video series.
2. CHW surveys: Post-intervention data were collected from consenting CHWs via an online Qualtrics survey administered in Spanish (the official written professional language used by CHWs). Participants were not compensated, as the goal was to assess interest in receiving and using voluntary educational materials absent financial incentives. The survey assessed beliefs about the safety, efficacy, and importance of routine and HPV vaccines, as well as perceived preparedness to address vaccine hesitancy, using a 4-point Likert scale (strongly disagree, somewhat disagree, somewhat agree, strongly agree). It also included a 10-item knowledge assessment drawn directly from CLAVE content. CHWs in the distribution arm were additionally asked to provide feedback on the usefulness and use of the materials in their work.

### Statistical analysis

We summarized social media analytics and survey data using descriptive statistics, and estimated intervention effects on CHW knowledge and perceptions with linear regression for continuous outcomes and mixed-effects logistic regression for binary outcomes. The primary exposure was assignment to the CHW-distribution arm (communities where CHWs directly received CLAVE content) versus the pooled social media–only and control arms. Standard errors were clustered at the department level for all models to account for intra-department correlation, and results for binary outcomes are reported as odds ratios with 95% confidence intervals. Analyses were conducted in Stata/SE 15 (StataCorp LLC, College Station, TX, USA).

### Reflexivity statement

This study was developed based on the extensive 15-year experience of Wuqu’ Kawoq | Maya Health Alliance, a community organization of Indigenous healthcare providers serving Indigenous populations. To foster a sense of safety, trust, and inclusiveness throughout the research process, Wuqu’ Kawoq Indigenous Maya staff actively participated in study design and implementation, leading ideation and review HCD sessions with Indigenous health workers and community members, reviewing and revising data collection protocols and instruments, and conducting IDIs and FGDs.

The research team also includes international researchers trained in community-based participatory research methods. To ensure the relevance of the study design and implementation and to address equity and ethical engagement with Indigenous communities, the team held weekly meetings with Wuqu’ Kawoq staff. Every member of the research team is dedicated to Wuqu’ Kawoq’s commitment to conducting research with leadership from members of the communities served. This commitment has fostered a community-engaged approach to our study design that persisted throughout the research process.

### Ethics approval

This project has been approved by the Institutional Review Boards at Stanford University (Protocol #63193), the University of California, San Francisco (Study #23-39043), and a local Guatemalan IRB through Wuqu’ Kawoq (Protocol #WK003). Informed consent was obtained from all participants prior to data collection. No members of the research team have financial interests in vaccine development or promotion.

## Results

### Social media reach and engagement

Table 1 summarizes CLAVE campaign performance metrics by video series and language over the six-week campaign period. The campaign reached nearly half a million unique users (486,594) across eight target communities, generating over 4.1 million impressions. Engagement was driven predominantly by video plays of three seconds or longer, followed by reactions, with shares, saves, and comments representing only a small proportion of total interactions. The aggregate engagement rate exceeded 100% (127.15%), reflecting that the engagement metric represents total interaction events rather than unique users and individual users may perform multiple interactions with the same content.

**Table 1:** Social media campaign reach, by video series and language

Video series, by language	Reach	Impressions	ThruPlays	ThruPlay rate	Cost (USD) per ThruPlay	Total engagements	Engagement rate	Engagements, by type					
								3-second plays	Reactions	Shares	Saves	Comments	Other
**Routine Immunization**	436,509	2,099,403	56,692	12.99%	$0.03	314,747	72.1%	312,370	2,063	180	96	12	26
Kaqchikel	333,558	892,226	24,790	7.43%	$0.02	134,594	40.35%	133,456	980	91	45	6	16
K’iche’	106,048	295,678	11,636	10.97%	$0.04	50,909	48.01%	50,401	436	45	21	4	2
Spanish	338,133	911,499	20,266	5.99%	$0.02	129,244	38.22%	128,513	647	44	30	2	8
**HPV**	432,600	2,088,919	53,507	12.37%	$0.03	303,942	70.26%	302,017	1,666	122	103	14	20
Kaqchikel	329,023	894,977	19,752	6.00%	$0.03	127,009	38.60%	126,244	669	40	40	7	9
K’iche’	106,860	300,284	11,921	11.16%	$0.04	51,027	47.75%	50,614	345	31	30	2	5
Spanish	333,583	893,658	21,834	6.55%	$0.02	125,906	37.74%	125,159	652	51	33	5	6
**Total**	486,594	4,188,322	110,199	22.65%	$0.03	618,689	127.15%	614,387	3,729	302	199	26	46

Notes: This table shows campaign performance metrics for the study period: reach (unique accounts that viewed the content); impressions (total on-screen displays, which may exceed reach due to repeated exposures); total engagements (sum of 3-second video plays, reactions, comments, shares, saves, and other engagements including link clicks to expand videos or text, profile clicks, profile clicks and other clicks on ad creative); and ThruPlays (number of times a video played for ≥15 seconds). Engagement rate was calculated as total engagements divided by reach, and ThruPlay rate as ThruPlays divided by reach. All metrics were aggregated for the campaign period and are reported stratified by language and video series

Spanish and Kaqchikel versions reached the largest audiences, each exceeding 300,000 unique users per series, while K’iche’ versions reached just over 100,000 users per series. Despite smaller reach, K’iche’ content consistently achieved the highest engagement and ThruPlay rates, reflecting proportionally greater interaction and video completion among those reached. Share rates remained low overall (<0.05%), but cost per ThruPlay was modest across all languages ($0.02–$0.04), demonstrating cost-efficient delivery. The combined reach across languages exceeded the total unique audience, indicating that many individuals were exposed to content in more than one language.

### Community health worker distribution and acceptability

We recruited 238 CHWs across 12 communities, of whom 156 consented to participate (55 in the CHW-distribution arm, 51 in the social media–only arm, and 50 in the control arm). Most participants were female (81%) and primarily ages 25–34 (44%) and 35–44 (31%). Language preferences were Spanish (81%), Kaqchikel (38%), and K’iche’ (8%). Nearly half reported serving more than 100 patients (47%), and most had experience administering vaccines (78%). Detailed CHW characteristics are provided in the Supplement.

As shown in Table 2, across all arms, vaccine acceptance among CHWs was high, with more than 80% of participants strongly agreeing with each of six vaccine confidence statements addressing the safety, efficacy, and importance of routine immunizations and the HPV vaccine. We observed no significant difference between the CHW arm and other arms for five of the confidence measures; however, CHWs receiving CLAVE videos directly were significantly more likely to strongly agree that the HPV vaccine is safe (*p* < 0.05). Across all arms, CHWs reported lower levels of vaccine acceptance among their patients compared to themselves. No significant differences were observed for CHW-reported patient vaccine acceptance statements.

**Table 2:** Community health worker (CHW) vaccine beliefs and knowledge

Outcomes	n (%)			Arm 1 vs. Arms 2 & 3 OR (95% CI)	p-value
	Arm 1: CHW + social media (N = 55)	Arm 2: social media (N = 51)	Arm 3: control (N = 50)		
**CHW vaccine beliefs**					
I think all childhood vaccines chosen for use in Guatemala are safe.	47 (85%)	41 (80%)	42 (84%)	1.27 (0.59, 2.75)	0.536
I think all childhood vaccines chosen for use in Guatemala are effective.	47 (85%)	43 (84%)	44 (88%)	0.95 (0.44, 2.02)	0.885
I think all childhood vaccines chosen for use in Guatemala are important to receive.	51 (93%)	42 (82%)	47 (94%)	1.72 (0.76, 3.89)	0.194
I think the HPV vaccine is safe.	52 (95%)	41 (80%)	43 (86%)	3.51 (1.27, 9.70)	0.016
I think the HPV vaccine is effective.	47 (85%)	39 (76%)	43 (86%)	1.36 (0.59, 3.14)	0.470
I think the HPV vaccine is important to receive.	52 (95%)	45 (88%)	44 (88%)	2.34 (0.32, 17.13)	0.404
**CHW preparedness**					
My patients trust and follow the health advice that I provide.	34 (62%)	35 (69%)	34 (68%)	0.75 (0.42, 1.35)	0.338
I feel prepared to answer questions from my patients.	40 (73%)	36 (71%)	33 (66%)	1.24 (0.62, 2.45)	0.543
I know who to ask when I don’t have the answers to questions my patients ask.	44 (80%)	41 (80%)	40 (80%)	0.99 (0.50, 1.96)	0.972
I have adequate access to information about vaccines and the diseases they prevent.	48 (87%)	39 (76%)	42 (84%)	1.69 (0.62, 4.63)	0.305
**CHW reports of patient vaccine beliefs**					
My patients believe that childhood vaccines are safe.	27 (49%)	34 (67%)	24 (48%)	0.71 (0.27, 1.87)	0.493
My patients know why childhood vaccines are important to receive.	34 (62%)	34 (67%)	31 (62%)	0.90 (0.31, 2.56)	0.839
My patients believe that the HPV vaccine is safe.	29 (53%)	22 (43%)	23 (46%)	1.39 (0.55, 3.50)	0.487
My patients believe that the HPV vaccine is important to receive.	30 (55%)	23 (45%)	24 (48%)	1.38 (0.58, 3.25)	0.464
**CHW vaccine knowledge**					
CHW knowledge score (out of 10), mean(SD)	7.69 (0.92)	7.80 (1.15)	7.84 (1.02)	–	0.739

Notes: This table shows the n (%) of CHWs in each study arm who strongly agree with survey statements admininstered at endline. The table also shows the show the mean and standard deviation of a 10-item knowledge assessment drawn directly from video content

Knowledge about vaccines among CHWs was also high across groups, with participants answering an average of 7.8 out of 10 knowledge questions correctly. We observed no significant difference in knowledge between arms. Across all arms, the majority of CHWs reported knowing where to turn when they did not have answers to patient questions, and reported feeling prepared to answer patient questions and felt they had adequate access to information about vaccines and the diseases they present.

In the four communities in the CHW information arm, 93% of participants reported that the routine immunization videos were helpful, and 91% reported that the HPV videos were helpful (see Table 3). Self-reported use of the videos with patients was high, with 95% of health workers stating they had used the routine immunization videos and 91% using the HPV videos in patient conversations. Nearly 90% of participants found the infographic job aids on both routine immunization and HPV to be helpful, and 91 and 93% reported using the routine immunization and HPV infographics, respectively, in discussions with patients.

**Table 3:** Community health worker (CHW) content feedback (*N* = 55)

Usefulness of content	Very helpful	A little helpful	Not helpful
How helpful were the videos on routine childhood vaccination for your work?	51 (93%)	4 (7%)	0 (0%)
How helpful are the static picture job aids on routine childhood vaccination for your work?	49 (89%)	6 (11%)	0 (0%)
How helpful were the videos on cervical cancer prevention and the HPV vaccine for your work?	50 (91%)	5 (9%)	0 (0%)
How helpful are the static picture job aids on cervical cancer prevention and the HPV vaccine for your work?	49 (89%)	6 (11%)	0 (0%)
**Utilization of content**	**Yes**	**No**	
Did you use the videos in your conversations with patients about routine childhood vaccination?	52 (95%)	3 (5%)	–
Did you use the static picture job aids in your conversations with patients about routine childhood vaccination?	50 (91%)	5 (9%)	–
Did you use the videos in your conversations with patients about cervical cancer prevention and the HPV vaccine?	50 (91%)	5 (9%)	–
Did you use the static picture job aids in your conversations with patients about cervical cancer prevention and the HPV vaccine?	51 (93%)	4 (7%)	–

Notes: This table shows the n (%) of the responses of CHWs in the CHW-distribution study arm in the endline survey

## Discussion

By averting an estimated 3.5–5 million deaths annually worldwide, routine vaccinations are one of the most effective public health interventions, and the ability to prevent disease and strengthen health systems resilience relies upon effective strategies to reach vulnerable populations with information regarding these life-saving measures [76]. Our study explores how a human-centered design approach that incorporates the partnership of community-based leaders, frontline providers, and community members into the design and distribution of vaccination education efforts can be deployed to effectively reach largely rural, Indigenous communities in Guatemala. This implementation study suggests that a multilingual, culturally tailored digital education campaign, delivered via geographically targeted paid social media and complemented by CHW job aids, can achieve broad exposure among social media audiences at low cost and be adopted by CHWs. Across eight communities, the CLAVE campaign reached nearly half a million unique users and generated over four million impressions over six weeks.

The aggregate engagement rate exceeded 100%, reflecting that the engagement metric captures total interaction events rather than unique users, and individual users may perform multiple interactions with the same content. Engagement was driven primarily by video plays of three seconds or longer, followed by far fewer reactions, while shares, saves, and comments represented only a very small fraction of total interactions. The very low share rates (<0.05%) indicate that nearly all exposure was driven by paid placement rather than organic diffusion of the content.

Although the campaign attained broad exposure, very few users chose to comment on the posts, with only 26 comments recorded across a reach of 486,594 unique users. The comments reflected a range of reactions, including expressions of appreciation, suggestions related to translation or language clarity, discussion of vaccine access, and some vaccine skepticism or misinformation. The low number of comments relative to the campaign reach may reflect the nature of paid social media dissemination, which can generate broad exposure but does not necessarily produce the same level of active interaction as organically shared content. As such, the campaign metrics primarily demonstrate the feasibility of reaching audiences through paid digital dissemination rather than evidence of sustained audience interaction with the content.

In addition to the social media dissemination pathway, we also examined the feasibility of distributing CLAVE materials through CHWs. Participating CHWs receiving CLAVE videos reported high rates of sharing with patients and positive feedback about the usefulness of the videos and job aids in their efforts to educate households in their care. These findings suggest that the intervention may be particularly valuable as a tool to support CHWs in their communication with households, complementing the broader exposure achieved through digital dissemination.

Our findings are consistent with prior evidence suggesting that culturally and linguistically health content may facilitate exposure to vaccine information and that trusted messengers like CHWs can support discussions about and engagement with health education materials [52]. The higher engagement and ThruPlay rates observed for K’iche’ content, despite smaller absolute reach, reflecting the smaller population of K’iche’ speakers in the study communities, suggest that materials developed through community-engaged design and local, less commonly spoken languages may resonate strongly in linguistic minority communities once users are reached. Conversely, broader reach for Spanish and Kaqchikel content is consistent with platform penetration patterns and likely reflects larger addressable audiences. Taken together, these findings highlight the potential for culturally and linguistically tailored content to reach audiences in underserved language communities when combined with deliberate digital placement strategies.

High adoption and reported use of materials by participating CHWs, combined with their already high vaccine confidence and knowledge, suggest that the incremental value of CHW-directed content in this pilot may be best suited for reinforcing preparedness and providing concrete communication tools rather than shifting CHW knowledge and perception. Across arms, baseline vaccine confidence and knowledge were already high, which likely limited detectable between-arm differences due to ceiling effects. The only significant difference observed was significantly higher agreement that the HPV vaccine is safe among the CHW-distribution arm. However, given greater apprehensions about the safety of the HPV vaccine in the focal communities [71], this finding may reflect a meaningful impact.

Because our findings are limited to CHWs who elected to participate, these results are not generalizable to all CHWs. Anecdotal feedback from Wuqu’ Kawoq implementers suggests that the low response rate was primarily attributable to time constraints, the light-touch nature of the intervention, and lack of incentives for participation. We conjecture that participating CHWs may have been more vaccine-acceptant, making it difficult to assess acceptability among less vaccine-acceptant CHWs. In addition, reliance on CHW self-reported measures introduces the possibility of social desirability bias. Future studies should build on these encouraging suggestive findings to engage a broader and more diverse sample of CHWs and examine impact on objective measures of video usage to address these limitations. Qualitative evidence on how CHWs utilize video content in their work could further inform the design and tailoring of video-based supports to better support CHW health education efforts.

The outcomes of this implementation study are also limited in their ability to speak to the downstream impact on vaccine knowledge and behaviors of community members. Measures of social media engagement are likewise limited in their ability to reflect meaningful engagement with CLAVE content as they capture interaction events rather than how viewers interpreted or responded to the information presented. In addition, the pilot did not include systematic data collection from community members regarding their perceptions of the intervention, limiting our ability to assess how the intended audience understood or responded to the content. In future research, we aim to leverage community-level health records data and survey data to examine the impact of the treatment arms on community attitudes and child vaccination rates. Such work will be important for assessing whether digital dissemination approaches like CLAVE translate into measurable improvements in vaccine knowledge or uptake.

As the Ministry of Health has indicated interest in scaling CLAVE nationally, broader impact may be evaluated using more rigorous rollout designs to understand the effectiveness of these efforts. At the same time, this pilot primarily demonstrates feasibility of dissemination and adoption by CHWs rather than proof of effectiveness in changing community-level attitudes or behaviors. Decisions about large-scale implementation should therefore be accompanied by continued evaluation and iterative refinement of the intervention as it expands.

We also note limitations in our human-centered design process that future studies can build upon to more fully capture community-level feedback on stigmatized topics. Although we collected in-depth input through gender-segregated focus group discussions, group-based methods may be influenced by social dynamics and may not fully capture individual perspectives on potentially sensitive topics, including HPV vaccination. While focus-group facilitators observed active participation and general comfort among participants in this setting and took steps to encourage broad input, we acknowledge the possibility of social desirability bias.

We also acknowledge that although community members participated in focus groups and provided feedback on prototype content, much of the iterative design input was provided through CHWs and partner organization staff. While these stakeholders have deep familiarity with community priorities and concerns, this approach may not fully capture the diversity of perspectives within the broader community. Moreover, because the pilot did not collect systematic feedback from community viewers following dissemination, engagement with the intended audience during the ADDIE evaluation phase was limited. Future work could benefit from deeper and more continuous engagement with community members across all stages of the design process. At the same time, the prominent role of CHWs in the design process reflects their central role as trusted intermediaries between health systems and Indigenous communities in Guatemala. Engaging CHWs as co-design partners allowed the intervention to incorporate insights from individuals who regularly communicate with families about vaccination and navigate community concerns, which is consistent with human-centered design approaches that incorporate both end-users and key service intermediaries.

Despite the limitations, more broadly, this pilot provides an example of a way in which academic institutions, NGOs, and ministries of health can collectively partner with local communities to co-create and implement a health education solution that is centered around the needs directly facing them. The study, co-led and implemented with members of the focal communities, explicitly centers Indigenous perspectives in defining their challenges, creating content, and evaluating intervention impacts. By addressing linguistic exclusion, contextual barriers, and mistrust, while leveraging technology in service of community priorities, this pilot illustrates how health services research can move beyond describing disparities to exploring community-designed solutions. The exposure achieved across multiple languages, underscores the potential value of culturally consonant approaches for populations historically excluded from mainstream health communication.

## Conclusions

This study explores the feasibility and potential role of community-engaged, linguistically and culturally tailored digital vaccination education in reaching audiences in rural Indigenous settings through paid social media dissemination and CHWs. The CLAVE campaign achieved broad exposure across multiple languages, while participating CHWs reported high acceptance of the materials and willingness to use the videos and job aids in their communication with households. Together, these findings suggest that tailored health education for Indigenous communities may strengthen existing immunization communication strategies when delivered through both digital platforms and trusted community intermediaries. Future research is needed to investigate the effectiveness of this and other such health education efforts on community-level perceptions and vaccine uptake.

## Electronic supplementary material

Below is the link to the electronic supplementary material.

Supplementary Material 1

## Data Availability

The datasets used and/or analysed during the current study are available from the corresponding author on reasonable request.

## Abbreviations

ADDIE: Analyze, Design, Develop, Implement, Evaluate
CHW/CHWs: Community health worker(s)
CI: Confidence interval
CLAVE: Culturally and Linguistically Appropriate Vaccine Education
COVID-19: Coronavirus disease 2019
DTP3: Third dose of diphtheria–tetanus–pertussis vaccine
FGD(s): Focus group discussion(s)
HCD: Human-centered design
HPV: Human papillomavirus
IRB: Institutional Review Board
LMIC(s): Low- and middle-income country(ies)
MMR2: Second dose of measles–mumps–rubella vaccine
MSPAS: Ministerio de Salud Pública y Asistencia Social (Guatemalan Ministry of Public Health and Welfare)
OR: Odds ratio
USD: United States dollars
WHO: World Health Organization

## References

[1] Guzman-Holst A, DeAntonio R, Prado-Cohrs D, Juliao P. Barriers to vaccination in Latin America: a systematic literature review. Vaccine. 2020;38(3):470–81. 31767469 10.1016/j.vaccine.2019.10.088

[2] PAHO/WHO-UNICEF Joint Reporting Forms (JRF). Vaccine coverage. 2023. https://ais.paho.org/imm/IM_JRF_COVERAGE.asp.

[3] Cata-Preta BDO, Wehrmeister FC, Santos TM, Barros AJD, Victora CG. Patterns in wealth-related inequalities in 86 low- and middle-income countries: global evidence on the emergence of vaccine hesitancy. Am J Prev Med. 2021;60(1):S24–33. 10.1016/j.amepre.2020.07.028 PMC7613086 33131990

[4] UNICEF. Country office annual report 2022 [Internet]. 2022. https://www.unicef.org/media/135941/file/Guatemala-2022-COAR.pdf.

[5] Pan American Health Organization (PAHO). Strategic Plan of the Pan American Health Organization 2014–2019 [Internet]. 2014. https://iris.paho.org/bitstream/handle/10665.2/7654/CD53-OD345-e.pdf?sequence=16%26isAllowed=y.

[6] World Health Organization (WHO). Immunization dashboard. 2022. https://immunizationdata.who.int/.

[7] Bevilacqua KG, Gottschlich A, Murchland AR, Alvarez CS, Rivera-Andrade A, Meza R. Cervical cancer knowledge and barriers and facilitators to screening among women in two rural communities in Guatemala: a qualitative study. BMC Womens Health. 2022;22(1):197. 35643497 10.1186/s12905-022-01778-y PMC9148459

[8] Sung H, Ferlay J, Siegel RL, Laversanne M, Soerjomataram I, Jemal A, et al. Global cancer statistics, 2020: GLOBOCAN estimates of incidence and mortality worldwide for 36 cancers in 185 countries. CA Cancer J Clin. 2021;71(3):209–49. 33538338 10.3322/caac.21660

[9] Bruni L, Albero G, Serrano B, Mena M, Collado J, Gomez D, et al. ICO/IARC information centre on HPV and cancer (HPV information centre). Human papillomavirus and related diseases in Guatemala. Summary Report. 2023.

[10] Skinner NA, Sanders K, Lopez E, Sotz Mux MS, Abascal Miguel L, Vosburg KB, et al. Barriers to COVID-19 vaccine acceptance to improve messages for vaccine uptake in indigenous populations in the central highlands of Guatemala: a participatory qualitative study. BMJ Open. 2023;13(1):e067210. 10.1136/bmjopen-2022-067210 PMC9884572 36707110

[11] Chary A, Flood D, Austad K, Colom M, Hawkins J, Cnop K, et al. Accompanying indigenous Maya patients with complex medical needs: a patient navigation system in rural Guatemala. Healthcare. 2018;6(2):144–49. 28919513 10.1016/j.hjdsi.2017.08.006

[12] International Work Group for Indigenous Affairs (IWGIA). Indigenous World, 2020: Guatemala [Internet]. 2020. https://www.iwgia.org/en/guatemala/3622-iw-2020-.

[13] Choudhary R, Carter E, Monzon J, Stewart A, Slotnick J, Samayoa Jerez LL, et al. Sociodemographic Factors associated with COVID-19 vaccination among people in Guatemalan municipalities. Nato Adv Sci Inst Se. 2023;11(4):745. 10.3390/vaccines11040745 PMC10143355 37112656

[14] Instituto Nacional de Estadística de Guatemala, INE. XII Censo Nacional de Población y VII de Vivienda 2018. 2018. https://censo2018.ine.gob.gt/.

[15] Ministry of Public Health and Social Welfare of the Republic of Guatemala (MSPAS). Vacunación de esquema regular en Guatemala [Internet]. 2023. Available from: https://tableros.mspas.gob.gt/vacunacionesquemaregular/.

[16] Taylor L. Guatemala’s COVID vaccine roll-out failed: here’s what researchers know. Nature. 2022. 10.1038/d41586-022-01804-x 35798864

[17] Abascal Miguel L, Lopez E, Sanders K, Skinner NA, Johnston J, Vosburg KB, et al. Evaluating the impact of a linguistically and culturally tailored social media ad campaign on COVID-19 vaccine uptake among indigenous populations in Guatemala: a pre/post design intervention study. BMJ Open. 2022;12(12):e066365. 10.1136/bmjopen-2022-066365 PMC9748511 36523220

[18] Flood D, Rohloff P. Indigenous languages and global health. Lancet Glob Health. 2018;6(2):e134–5. 10.1016/S2214-109X(17)30493-X 29389530

[19] Wong M, Haswell-Elkins M, Tamwoy E, McDermott R, d’Abbs P. Perspectives on clinic attendance, medication and foot-care among people with diabetes in the Torres Strait Islands and Northern Peninsula Area. Aust J Rural Health. 2005;13(3):172–77. 15932487 10.1111/j.1440-1854.2005.00678.x

[20] Al Shamsi H, Almutairi AG, Al Mashrafi S, Al Kalbani T. Implications of language barriers for healthcare: a systematic review. Oman Med J. 2020;35(2):e122–122. 10.5001/omj.2020.40 PMC7201401 32411417

[21] Lowell A, Devlin B. Miscommunication between aboriginal students and their non-aboriginal teachers in a bilingual school. Lang Cult Curric. 1998;11(3):367–89.

[22] De Moissac D, Bowen S. Impact of language barriers on quality of care and patient safety for official language minority francophones in Canada. J Patient Exp. 2019;6(1):24–32. 31236448 10.1177/2374373518769008 PMC6572938

[23] Karliner LS, Kim SE, Meltzer DO, Auerbach AD. Influence of language barriers on outcomes of hospital care for general medicine inpatients. J Hosp Med. 2010;5(5):276–82. 20533573 10.1002/jhm.658

[24] Cohen AL, Rivara F, Marcuse EK, McPhillips H, Davis R. Are language barriers associated with serious medical events in hospitalized pediatric patients? Pediatrics. 2005;116(3):575–79. 16140695 10.1542/peds.2005-0521

[25] Divi C, Koss RG, Schmaltz SP, Loeb JM. Language proficiency and adverse events in US hospitals: a pilot study. Int J Qual Health Care. 2007;19(2):60–67. 17277013 10.1093/intqhc/mzl069

[26] Foo PK, Perez B, Gupta N, Lorenzo GJ, Misa NY, Gutierrez BS, et al. High rates of COVID-19 infection among Indigenous Maya at a US safety-net health system in California. Public Health Rep. 2021;136(3):295–300. 33593141 10.1177/0033354921990370 PMC8580403

[27] Mora AM, Lewnard JA, Kogut K, Rauch SA, Hernandez S, Wong MP, et al. Risk factors associated with SARS-CoV-2 infection among farmworkers in Monterey County, California. JAMA Netw Open. 2021;4(9):e2124116. 10.1001/jamanetworkopen.2021.24116 PMC8444020 34524438

[28] Anderson I, Robson B, Connolly M, Al-Yaman F, Bjertness E, King A, et al. Indigenous and tribal peoples’ health (the Lancet-Lowitja Institute Global Collaboration): a population study. Lancet. 2016;388(10040):131–57. 27108232 10.1016/S0140-6736(16)00345-7

[29] Gracey M, King M. Indigenous health part 1: determinants and disease patterns. Lancet. 2009;374(9683):65–75. 19577695 10.1016/S0140-6736(09)60914-4

[30] Wilson E, Hm Chen A, Grumbach K, Wang F, Fernandez A. Effects of limited English proficiency and physician language on health care comprehension. J Gen Intern Med. 2005;20(9):800–06. 16117746 10.1111/j.1525-1497.2005.0174.x PMC1490205

[31] Artuso S, Cargo M, Brown A, Daniel M. Factors influencing health care utilisation among Aboriginal cardiac patients in central Australia: a qualitative study. BMC Health Serv Res. 2013;13(1):83. 23497140 10.1186/1472-6963-13-83 PMC3606832

[32] Shahid S, Finn LD, Thompson SC. Barriers to participation of Aboriginal people in cancer care: communication in the hospital setting. Med J Aust. 2009;190(10):574–79. 19450207 10.5694/j.1326-5377.2009.tb02569.x

[33] Webster P. Language barriers restricting access to health care for Indigenous populations. Can Med Assoc J. 2018;190(24):E754–5. 10.1503/cmaj.109-5613 PMC6008191 29914917

[34] Yashadhana A, Fields T, Blitner G, Stanley R, Zwi AB. Trust, culture and communication: determinants of eye health and care among Indigenous people with diabetes in Australia. BMJ Glob Health. 2020;5(1):e001999. 10.1136/bmjgh-2019-001999 PMC7042588 32133172

[35] Poirier B, Sethi S, Garvey G, Hedges J, Canfell K, Smith M, et al. HPV vaccine: uptake and understanding among global Indigenous communities - a qualitative systematic review. BMC Public Health. 2021;21(1):2062. 34758805 10.1186/s12889-021-12147-z PMC8582096

[36] Mosby I, Swidrovich J. Medical experimentation and the roots of COVID-19 vaccine hesitancy among Indigenous Peoples in Canada. Can Med Assoc J. 2021;193(11):E381–3. 10.1503/cmaj.210112 PMC8096406 33627413

[37] Driedger SM, Maier R, Furgal C, Jardine C. Factors influencing H1N1 vaccine behavior among Manitoba Metis in Canada: a qualitative study. BMC Public Health. 2015;15(1):128. 25884562 10.1186/s12889-015-1482-2 PMC4334920

[38] Beck E. The uneven impacts of violence against women reform in Guatemala: intersecting inequalities and the patchwork state. Lat Am Res Rev. 2021;56(1):20–35.

[39] Meneses-Navarro S, Freyermuth-Enciso MG, Pelcastre-Villafuerte BE, Campos-Navarro R, Meléndez-Navarro DM, Gómez-Flores-Ramos L. The challenges facing indigenous communities in Latin America as they confront the COVID-19 pandemic. Int J Equity Health. 2020;19(1):63. 32381022 10.1186/s12939-020-01178-4 PMC7203711

[40] Sanford V. From genocide to feminicide: impunity and human rights in twenty-first century Guatemala. J Hum Rights. 2008;7(2):104–22.

[41] Cerón A, Ruano AL, Sánchez S, Chew AS, Díaz D, Hernández A, et al. Abuse and discrimination towards indigenous people in public health care facilities: experiences from rural Guatemala. Int J Equity Health. 2016;15(1):77. 27177690 10.1186/s12939-016-0367-z PMC4866428

[42] Perry H, Crigler L, Hodgins S. Developing and strengthening community health worker programs at scale: a reference guide for program managers and policy makers. Dhaka, Bangladesh: University Press Ltd; 2013.

[43] Nzioki JM, Ouma J, Ombaka JH, Onyango RO. Community health worker interventions are key to optimal infant immunization coverage, evidence from a pretest-posttest experiment in Mwingi, Kenya. Pan Afr Med J. 2017;28(1). 10.11604/pamj.2017.28.21.11255 PMC5680999 29138657

[44] Freeman PA, Schleiff M, Sacks E, Rassekh BM, Gupta S, Perry HB. Comprehensive review of the evidence regarding the effectiveness of community-based primary health care in improving maternal, neonatal and child health: 4. child health findings. J Global Health. 2017;7(1):010904. 10.7189/jogh.07.010904 PMC5491948 28685042

[45] Callaghan-Koru JA, Gilroy K, Hyder AA, George A, Nsona H, Mtimuni A, et al. Health systems supports for community case management of childhood illness: lessons from an assessment of early implementation in Malawi. BMC Health Serv Res. 2013;13(1):55. 23394591 10.1186/1472-6963-13-55 PMC3637472

[46] Dahn B, Woldermariam AT, Perry H, et al. Strengthening primary health care through community health workers: investment case and financing recommendations. 2015.

[47] Glenton C, Javadi D, Perry HB. Community health workers at the dawn of a new era: 5. Roles and tasks. Health Res Policy Syst. 2021;19(3):128. 34641903 10.1186/s12961-021-00748-4 PMC8506082

[48] Lazarus JV, Wyka K, White TM, Picchio CA, Rabin K, Ratzan SC, et al. Revisiting COVID-19 vaccine hesitancy around the world using data from 23 countries in 2021. Nat Commun. 2022;13(1):3801. 35778396 10.1038/s41467-022-31441-x PMC9247969

[49] Do Nascimento IJ, Pizarro AB, Almeida JM, Azzopardi-Muscat N, Gonçalves MA, Björklund M, et al. Infodemics and health misinformation: a systematic review of reviews. Bull World Health Organ. 2022;100(9):544. 36062247 10.2471/BLT.21.287654 PMC9421549

[50] Wilson SL, Wiysonge C. Social media and vaccine hesitancy. BMJ Global Health. 2020;5(10). 10.1136/bmjgh-2020-004206 PMC7590343 33097547

[51] Ruggeri K, Vanderslott S, Yamada Y, Argyris YA, Većkalov B, Boggio PS, et al. Behavioural interventions to reduce vaccine hesitancy driven by misinformation on social media. BMJ. 2024;384. 10.1136/bmj-2023-076542 PMC10789192 38228339

[52] Abascal Miguel L, Lopez E, Sanders K, Skinner NA, Johnston J, Vosburg KB, et al. Evaluating the impact of a linguistically and culturally tailored social media ad campaign on COVID-19 vaccine uptake among indigenous populations in Guatemala: a pre/post design intervention study. BMJ Open. 2022;12(12):e066365. 10.1136/bmjopen-2022-066365 PMC9748511 36523220

[53] Jones R, Crowshoe L, Reid P, Calam B, Curtis E, Green M, et al. Educating for Indigenous health equity: an international consensus statement. Acad Med. 2019;94(4):512–19. 30277958 10.1097/ACM.0000000000002476 PMC6445615

[54] Melles M, Albayrak A, Goossens R. Innovating health care: key characteristics of human-centered design. Int J Qual Health Care. 2021;33(Suppl_1):37–44. 33068104 10.1093/intqhc/mzaa127 PMC7802070

[55] Bazzano AN, Martin J, Hicks E, Faughnan M, Murphy L. Human-centred design in global health: a scoping review of applications and contexts. Virgili G, editor. PLoS One. 2017;12(11):e0186744. 10.1371/journal.pone.0186744 PMC5665524 29091935

[56] MacDonald NE. Vaccine hesitancy: definition, scope and determinants. Vaccine. 2015;33(34):4161–64. 25896383 10.1016/j.vaccine.2015.04.036

[57] Poland CM, Matthews AKS, Poland GA. Improving COVID-19 vaccine acceptance: including insights from human decision-making under conditions of uncertainty and human-centered design. Vaccine. 2021;39(11):1547–50. 33612343 10.1016/j.vaccine.2021.02.008 PMC7875011

[58] Salmon DA, Dudley MZ, Glanz JM, Omer SB. Vaccine Hesitancy. Am J Prev Med. 2015;49(6):S391–8. 10.1016/j.amepre.2015.06.009 26337116

[59] Adam M, Tomlinson M, Le Roux I, LeFevre AE, McMahon SA, Johnston J, et al. The Philani MOVIE study: a cluster-randomized controlled trial of a mobile video entertainment-education intervention to promote exclusive breastfeeding in South Africa. BMC Health Serv Res. 2019;19(1):211. 30940132 10.1186/s12913-019-4000-x PMC6444854

[60] Adam M, Johnston J, Job N, Dronavalli M, Le Roux I, Mbewu N, et al. Evaluation of a community-based mobile video breastfeeding intervention in Khayelitsha, South Africa: the Philani MOVIE cluster-randomized controlled trial. Bassat Q, editor. PLoS Med. 2021;18(9):e1003744. 10.1371/journal.pmed.1003744 PMC8478218 34582438

[61] Isler J, Sawadogo NH, Harling G, Bärnighausen T, Adam M, Sié A, et al. ‘If he sees it with his own eyes, he will understand’: how gender informed the content and delivery of a maternal nutrition intervention in Burkina Faso. Health Policy Plan. 2020;35(5):536–45. 32106288 10.1093/heapol/czaa012 PMC7225566

[62] Isler J, Sawadogo NH, Harling G, Bärnighausen T, Adam M, Kagoné M, et al. Iterative adaptation of a maternal nutrition videos mHealth intervention across countries using human-centered design: qualitative study. JMIR MHealth UHealth. 2019;7(11):e13604. 10.2196/13604 PMC6878105 31710302

[63] Chamberlain AT, Limaye RJ, O’Leary ST, Frew PM, Brewer SE, Spina CI, et al. Development and acceptability of a video-based vaccine promotion tutorial for obstetric care providers. Vaccine. 2019;37(19):2532–36. 30962093 10.1016/j.vaccine.2019.03.005 PMC6472923

[64] Muinga N, Paton C, Gicheha E, Omoke S, Abejirinde IOO, Benova L, et al. Using a human-centred design approach to develop a comprehensive newborn monitoring chart for inpatient care in Kenya. BMC Health Serv Res. 2021;21(1):1010. 34556098 10.1186/s12913-021-07030-x PMC8461871

[65] Shearer JC, Nava O, Prosser W, Nawaz S, Mulongo S, Mambu T, et al. Uncovering the drivers of childhood immunization inequality with caregivers, community members and health system stakeholders: results from a human-centered design study in DRC, Mozambique and Nigeria. Nato Adv Sci Inst Se. 2023;11(3):689. 10.3390/vaccines11030689 PMC10054670 36992273

[66] McKinnon B, Abalovi K, Vandermorris A, Dubé È, Tuong Nguyen C, Billou N, et al. Using human-centred design to tackle COVID-19 vaccine hesitancy for children and youth: a protocol for a mixed-methods study in Montreal, Canada. BMJ Open. 2022;12(4):e061908. 10.1136/bmjopen-2022-061908 PMC8983461 35383090

[67] Henninger ML, McMullen CK, Firemark AJ, Naleway AL, Henrikson NB, Turcotte JA. User-centered design for developing interventions to improve clinician recommendation of human papillomavirus vaccination. Perm J. 2017;21(4):16–191. 28898195 10.7812/TPP/16-191 PMC5593511

[68] Reñosa MDC, Wachinger J, Guevarra JR, Landicho-Guevarra J, Aligato MF, Endoma V, et al. Human-centred design bolsters vaccine confidence in the Philippines: results of a randomised controlled trial. BMJ Glob Health. 2023;8(10):e012613. 10.1136/bmjgh-2023-012613 PMC10603469 37865401

[69] Branson RK, Rayner GT, Cox JL, Furman JP, King FJ. Interservice procedures for instructional systems development. (TRADOC Pam 350–30 NAVEDTRA 106A), Vol. 5. Florida State University Tallahassee Center for Educational Technology; 1975.

[70] Schlegel M. A handbook of instructional and training program design [Internet]. 1995. https://files.eric.ed.gov/fulltext/ED383281.pdf.

[71] Skinner NA, Abascal-Miguel L, Lopez E, Sotz M, Coxaj J, Ward V, et al. ‘How can I explain it to my daughter if I don’t know?’: understanding health communication and routine and HPV vaccine hesitancy in rural Indigenous communities of Guatemala. medRxiv. 2025:2025–28.

[72] ITU. Percentage of population using the internet in Guatemala from 2010 to 2021 [Graph]. In: Statista. 2023. https://www-statista-com.stanford.idm.oclc.org/statistics/1055473/internet-penetration-guatemala/.

[73] DataReportal. Digital 2023: Guatemala - global digital insights [Internet]. https://datareportal.com/reports/digital-2023-guatemala.

[74] iLifebelt (@iLifebelt). Departments with the highest Facebook penetration rate in Guatemala in 2020 [Graph]. In: Statista. https://www-statista-com.stanford.idm.oclc.org/statistics/1200073/departments-highest-percentage-facebook-users-guatemala/.

[75] Meta Business Help Center. About ThruPlay [Internet]. Menlo Park (CA): Meta Platforms, Inc. https://www.facebook.com/business/help/2051461368219124.

[76] World Health Organization. Vaccines and immunization [Internet]. Geneva (CH): WHO. https://www.who.int/health-topics/vaccines-and-immunization.

