# Supplementary material for: Culturally and linguistically appropriate vaccine education (CLAVE) for Indigenous communities: design and implementation study of a community-based approach to digital vaccine education in the Central Highlands of Guatemala
Source: Int J Equity Health. 2026 Apr 14;25:113. doi: 10.1186/s12939-026-02836-9 (PMC13126958; doi:10.1186/s12939-026-02836-9)
Supplement: Supplementary file 1 — Supplementary Material 1 [file 12939_2026_2836_MOESM1_ESM.pdf]

Supplement Table 1 . Length of CLAVE videos, by topic area and language			
	Length (minutes:seconds)		
**Topic Area**	**Kaqchikel**	**K'iche'**	**Spanish**
**Routine Immunization**			
Video 1: Immunization Schedule and Importance	01:42	01:53	01:35
Video 2: How Vaccines Work and Side Effects	01:22	01:09	01:05
Video 3: Addressing Misinformation and Sources of Hesitancy	01:35	01:38	01:14
**HPV**			
Video 1: HPV Connection to Cancer	01:18	01:24	00:59
Video 2: Myths and Misinformation	01:29	01:57	01:17
Video 3: HPV Vaccine Schedule	01:29	01:55	01:12

Supplement Table 2 . Community health worker (CHW) baseline characteristics				
**Characteristics**	**Total (N=156)**	**Arm 1: CHW + social media (N=55)**	**Arm 2: social media (N=51)**	**Arm 3: control (N=50)**
**Age**				
18-24 years	16 (10%)	5 (9%)	9 (18%)	2 (4%)
25-34 years	69 (44%)	22 (40%)	25 (49%)	22 (44%)
35-44 years	49 (31%)	19 (35%)	11 (22%)	19 (38%)
45-60 years	19 (12%)	7 (13%)	6 (12%)	6 (12%)
Older than 60 years	3 (2%)	2 (4%)	0 (0%)	1 (2%)
**Gender**				
Female	127 (81%)	45 (82%)	43 (84%)	39 (78%)
Male	29 (19%)	10 (18%)	8 (16%)	11 (22%)
**Preferred language(s)**				
Kaqchikel	59 (38%)	23 (42%)	14 (27%)	22 (44%)
K'iche'	12 (8%)	4 (7%)	7 (14%)	1 (2%)
Spanish	127 (81%)	47 (85%)	43 (84%)	37 (74%)
**Years of CHW experience**				
Less than 1 year	42 (27%)	14 (25%)	18 (35%)	10 (20%)
1-3 years	23 (15%)	10 (18%)	6 (12%)	7 (14%)
4-5 years	32 (21%)	12 (22%)	10 (20%)	10 (20%)
6-10 years	40 (26%)	12 (22%)	12 (24%)	16 (32%)
11-20 years	3 (2%)	0 (0%)	1 (2%)	2 (4%)
More than 20 years	16 (10%)	7 (13%)	4 (8%)	5 (10%)
**Experience giving vaccines**				
No	34 (22%)	21 (38%)	4 (8%)	9 (18%)
Yes	122 (78%)	34 (62%)	47 (92%)	41 (82%)
**Number of patients**				
Fewer than 10 patients	25 (16%)	12 (23%)	5 (10%)	8 (16%)
11-25 patients	19 (12%)	11 (21%)	5 (10%)	3 (6%)
26-50 patients	20 (13%)	7 (13%)	7 (14%)	6 (12%)
51-100 patients	18 (12%)	4 (8%)	7 (14%)	7 (14%)
More than 100 patients	72 (47%)	19 (36%)	27 (53%)	26 (52%)
**Patients' preferred language(s)**				
Kaqchikel	99 (63%)	42 (76%)	23 (45%)	34 (68%)
K'iche'	13 (8%)	1 (2%)	11 (22%)	1 (2%)
Spanish	118 (76%)	44 (80%)	36 (71%)	38 (76%)
**Primary location of patients**				
Peri-urban	71 (46%)	30 (56%)	22 (43%)	19 (38%)
Rural	68 (44%)	18 (33%)	20 (39%)	30 (60%)
Urban	16 (10%)	6 (11%)	9 (18%)	1 (2%)

NOTES: This table shows the n(%) of CHWs in each study arm with the listed characteristics. CHWs could report more than one preferred language for themselves and their patience. Hence percentages exceed 100%.
